# Genome-Wide Characterization and Identification of Long Non-Coding RNAs during the Molting Process of a Spider Mite, *Panonychus citri*

**DOI:** 10.3390/ijms22136909

**Published:** 2021-06-27

**Authors:** Gang Li, Xunyan Liu, Guy Smagghe, Jinzhi Niu, Jinjun Wang

**Affiliations:** 1 Provincial Key Laboratory of Agricultural Pest Management of Mountainous Regions, Institute of Entomology, Guizhou University, Guiyang 550025, China; agrgli@163.com; 2 International Joint Laboratory of China-Belgium on Sustainable Crop Pest Control, State Cultivation Base of Crop Stress Biology for Southern Mountainous Land, Academy of Agricultural Sciences, Southwest University, Chongqing 400715, China; liuxy1683@163.com (X.L.); Guy.Smagghe@UGent.be (G.S.); jinzhiniu@swu.edu.cn (J.N.); 3 Department of Plants and Crops, Faculty of Bioscience Engineering, Ghent University, 9000 Ghent, Belgium

**Keywords:** *Panonychus citri*, long non-coding RNA, molting process, hub lncRNA

## Abstract

Molting is essential for arthropods to grow. As one of the important arthropod pests in agriculture, key spider mite species (*Tetranychus* and *Panonychus*) can normally molt three times from the larva to adult stage within a week. This physiological strategy results in the short lifecycle of spider mites and difficulties in their control in the field. Long non-coding RNAs (lncRNAs) regulate transcriptional editing, cellular function, and biological processes. Thus, analysis of the lncRNAs in the spider mite molting process may provide new insights into their roles in the molting mechanism. For this purpose, we used high-throughput RNA-seq to examine the expression dynamics of lncRNAs and mRNAs in the molting process of different development stages in *Panonychus citri*. We identified 9199 lncRNAs from 18 transcriptomes. Analysis of the lncRNAs suggested that they were shorter and had fewer exons and transcripts than mRNAs. Among these, 356 lncRNAs were differentially expressed during three molting processes: late larva to early protonymph, late protonymph to early deutonymph, and late deutonymph to early adult. A time series profile analysis of differentially expressed lncRNAs showed that 77 lncRNAs were clustered into two dynamic expression profiles (Pattern a and Pattern c), implying that lncRNAs were involved in the molting process of spider mites. Furthermore, the lncRNA–mRNA co-expression networks showed that several differentially expressed hub lncRNAs were predicted to be functionally associated with typical molting-related proteins, such as cuticle protein and chitin biosynthesis. These data reveal the potential regulatory function of lncRNAs in the molting process and provide datasets for further analysis of lncRNAs and mRNAs in spider mites.

## 1. Introduction

Long non-coding RNAs (lncRNAs) are more than 200 bp in length and lack coding capability [1]. As with mRNAs, lncRNAs are transcribed by RNA polymerase II through splicing, capping, and polyadenylation [2]. Compared with mRNAs, lncRNAs have lower expression and sequence conservation, so lncRNAs are hard to identify and annotate based on the conserved sequences [3,4]. The lncRNAs are divided into four categories (sense, antisense, intronic, and intergenic) based on their position and direction of transcription about the protein-coding genes [5]. Sequencing technology has identified abundant lncRNAs in some species and verified lncRNAs to be important regulators of biological processes rather than simply transcription byproducts [6,7,8,9,10]. lncRNAs could regulate the expression of protein-coding genes by *cis*-regulation of near genes and *trans*-regulation of terminal genes in multiple biological processes, such as mRNA transcription, post-transcription, and stability [11,12]. 

lncRNAs have been identified in *Sogatella frucifera* [8], *Tribolium castaneum* [9], *Drosophila melanogaster* [13], *Locusta migratoria* [14], *Apis cerana* [15], *Plutella xylostella* [16], and *Bombyx mori* [17]. The functional annotation of lncRNAs in insect species has revealed that they can regulate immunity, behavioral plasticity, development, fecundity, and resistance [18,19,20,21,22]. For example, lncRNAs were identified in different developmental stages of *T. castaneum*. These results suggested that lncRNAs could act on the metabolic enzymes related to development [9].

Spider mites are agricultural pests that widely damage plants. They are resistant to acaricides within 2–4 years, and this is partially due to their short generation time [23,24,25,26,27]. For instance, *Panonychus citri* can complete its lifecycle, including the egg, larva, protonymph, deutonymph, and adult stages, in 14 d. During development, *P. citri* needs to molt three times. The molting process of spider mites regulated by the ecdysteroid Ponasterone A has been reported in previous studies involving ecdysteroid pathway-related genes [28,29,30,31,32]. Although lncRNAs have been characterized in insects, there are few studies on the identification and functional annotation of lncRNAs in mites. In *Varroa destructor*, 6645 novel lncRNA transcripts were identified by RNA-seq, and function enrichment analysis predicted that these lncRNAs could regulate the reproductive phase of *V. destructor* [33]. In *Tetranychus cinnabarinus*, 4454 lncRNAs were identified in a cyflumetofen-resistant strain, and *lincRNA_Tc13743.2* could decrease the inhibitory function of miR-133-5p that affected the expression of *TcGSTm02* to regulate cyflumetofen resistance [34]. No lncRNA study has shown that lncRNAs regulate the molting process in spider mites. *P. citri* is an economically important pest in citrus. This mite feeds on leaves, fruits, and green twigs by piercing and sucking. We performed RNA-seq to systematically identify the lncRNA transcripts, characterize the lncRNAs expression, and annotate their functions in three molting processes from larva to adult. We identified several lncRNA candidates for characterization and potential lncRNA regulation targets during the molting process of *P. citri*.

## 2. Results

### 2.1. Identification and Characterization of lncRNAs

**Figure 1:**
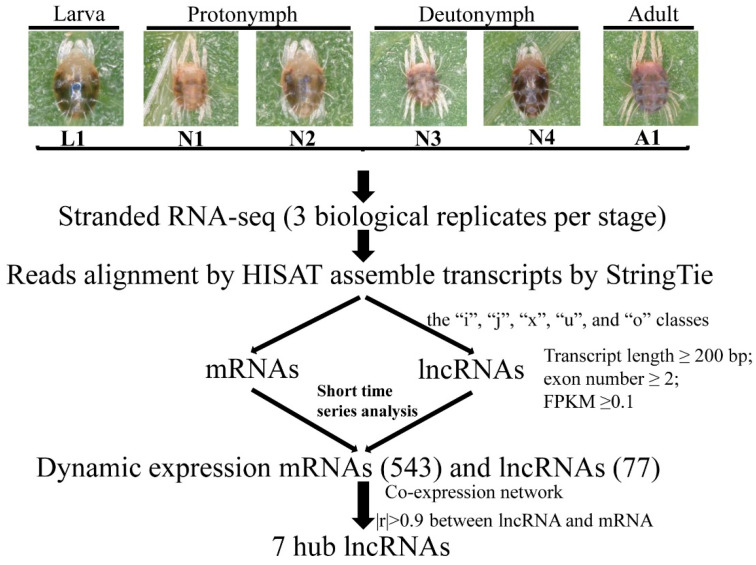
The experimental synthesis outline for sampling and sequencing in the molting process of different development stages.

**Figure 2:**
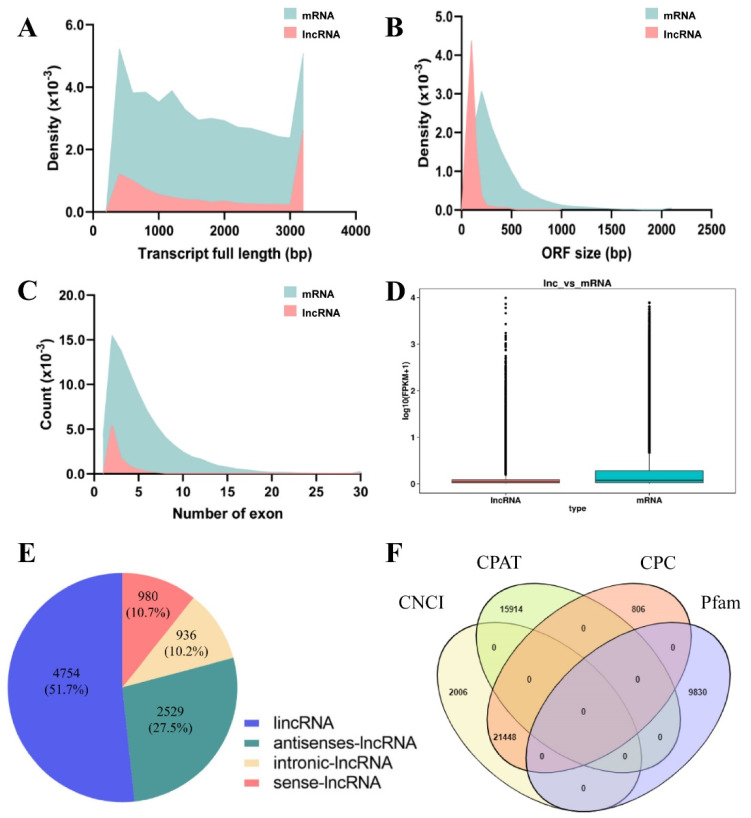
Identification and characterization of lncRNAs in *Panonychus citri.* (**A**) Full length distribution of lncRNAs and mRNAs in the mite *P. citri*. (**B**) Maximum open reading frame (ORF) size distribution of lncRNAs and mRNAs in the mite *P. citri*. (**C**) Number of exons per transcript for lncRNAs and mRNAs. (**D**) Overall expression (log_2_(FPKM+1)) of lncRNAs compared with mRNAs in *P. citri*. (**E**) lncRNA categories and their proportions. (**F**) Venn diagram of lncRNAs by different prediction methods. CNCI: Coding-Non-Coding-Index; CPAT: Coding Potential Assessment Tool; CPC: Coding Potential Calculator; Pfam: Pfam database.

To systematically identify the lncRNAs involved in molting process in *P. citri*, 18 RNA libraries with three biological replications for each molting time point were constructed by the Illumina Hiseq 3000 platform (Figure 1). After filtering reads containing poly-N, adapter reads, and low-quality reads, 369.35 Gb of clean data was obtained from 18 samples in total, and the Q30 was evaluated to be higher than 95.18%. The GC content in all samples was 39.0% on average. Next, clean reads were aligned with the genome database of *P. citri* (https://bioinformatics.psb.ugent.be/orcae/overview/Panci, accessed on 22 June 2021). The mapped rate of the 18 samples ranged from 74.61% to 92.79% (Table S1). In total, 12,955 known mRNAs were obtained from the transcripts based on the spider mite genome sequences, and 9199 highly reliable putative lncRNAs were identified from the unknown transcripts (Figure 2F). Compared with the genomic features of assembled known mRNAs, the putative lncRNAs lengths were between 202 bp and 44,932 bp, and the median length was 1528 bp shorter than the 3252 bp found in the mRNAs (Figure 2A). The size of the lncRNA open reading frame was also shorter than that of the mRNAs (Figure 2B). For the number of exons, 60% of the lncRNAs included two exons, while the mRNAs’ exons ranged from 1 to 63 (Figure 2C). The overall expression level of mRNAs was higher than that of lncRNAs (Figure 2D). The lncRNAs were classified as lincRNA (4754, 51.7%), intronic lncRNA (936, 10.2%), sense lncRNA (980, 10.7%), and antisense lncRNA (2529, 27.5%) based on the lncRNAs’ genomic locations relative to the mRNAs (Figure 2E). 

### 2.2. Differential Expression of lncRNAs in the Molting Process of Different Development Stages

**Figure 3:**
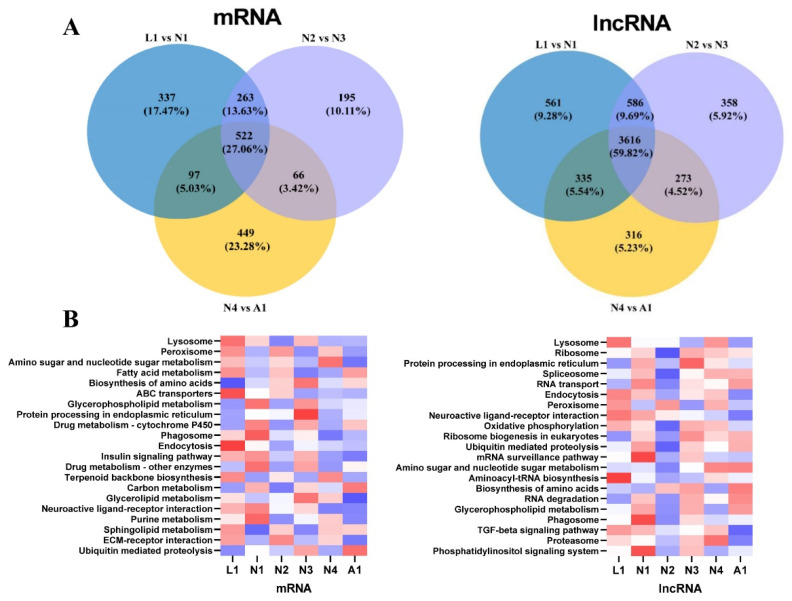
Expressed lncRNAs and mRNAs in the molting processes of different development stages in *Panonychus citri*. (**A**) Venn diagram of expressed lncRNAs and mRNAs in the molting processes of different development stages. (**B**) The top 21 enriched Kyoto Encyclopedia of Genes and Genomes (KEGG) pathways of long non-coding RNA (lncRNA) targets and mRNA transcripts in the molting processes of different development stages. L1: late larva; N1: early protonymph; N2: late protonymph; N3: early deutonymph; N4: late deutonymph; A1: early adult.

In total, 6045 lncRNAs were found to be expressed during the molting process of the different developmental stages. Of these lncRNAs, 3616 (59.82%) lncRNAs were co-expressed in the three molting processes from the larva to adult stages, and 561 (9.28%) were expressed specifically in the molting process from larva to protonymph, 358 (5.92%) from protonymph to deutonymph, and 316 (5.23%) from deutonymph to adult (Figure 3A, right). In total, 1929 mRNAs were expressed in the molting processes of different developmental stages, while 522 (27.06%) mRNAs were co-expressed in three molting processes from larva to adult, and 337 (17.47%) were specifically expressed in the molting process from larva to protonymph, 195 (10.11%) from protonymph to deutonymph, and 449 (23.28%) from deutonymph to adult (Figure 3A, left). KEGG enrichment analysis of the expression of all transcripts showed that 923 lncRNAs were significantly enriched in 144 pathways (Table S2), including the lysosome, ribosome, protein processes in the endoplasmic reticulum, spliceosome, RNA transport, and endocytosis pathways, whereas 629 mRNAs were significantly enriched in 131 pathways (Table S2), including the lysosome, peroxisome, amino sugar and nucleotide sugar metabolism, fatty acid metabolism, biosynthesis of amino acid, and ABC transport pathways. These results suggested that lncRNAs regulated the biological pathways differently from mRNAs (Figure 3B). 

**Figure 4:**
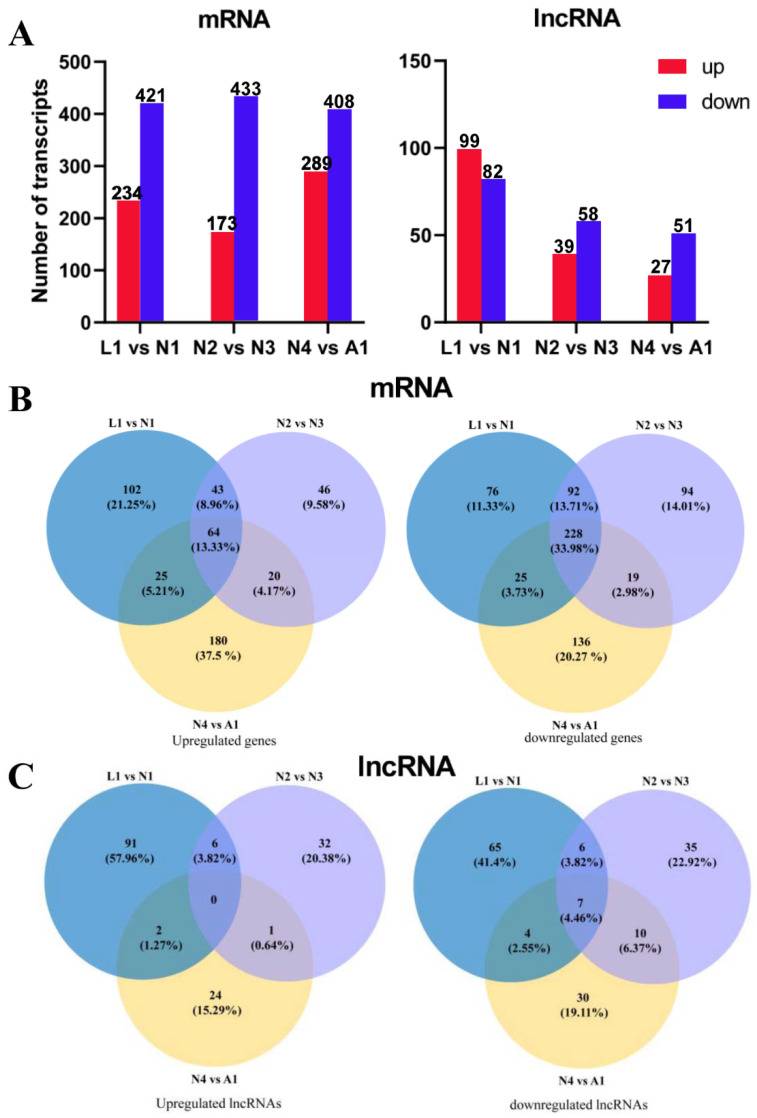
DE lncRNAs and mRNAs in the molting processes of different development stages in *Panonychus citri*. (**A**) Up- and downregulated lncRNAs and mRNAs in the molting processes of different development stages. Venn diagram of up- and downregulated differentially expressed mRNAs (**B**) and lncRNAs (**C**) in the molting processes of different development stages. L1: late larva; N1: early protonymph; N2: late protonymph; N3: early deutonymph; N4: late deutonymph; A1: early adult.

To screen the lncRNAs with differential expression during the molting process, 356 lncRNAs in total were expressed differentially in the molting processes of different developmental stages, whereas 1958 mRNAs were expressed differentially. Of these genes, 696 mRNAs and 165 lncRNAs were upregulated, whereas 1262 mRNAs and 191 lncRNAs were downregulated (log_2_FC ≥ 1.0 and *p*-value < 0.05) (Figure 4A). For the differential expression of lncRNAs, there was no co-expressed lncRNAs upregulated, but 91, 32, and 24 lncRNAs were upregulated and expressed specifically in the molting process from larva to protonymph (L1 vs. N1), protonymph to deutonymph (N2 vs. N3), and deutonymphs to adult (N4 vs. A1), respectively. There were seven lncRNAs co-expressed and downregulated in three molting processes of different developmental stages, and 65, 32, and 30 lncRNAs were downregulated and expressed specifically in the molting process from larva to protonymph (L1 vs. N1), protonymph to deutonymph (N2 vs. N3), and deutonymph to adult (N4 vs. A1), respectively (Figure 4C). There were 64 mRNAs with co-upregulated expression and 228 mRNAs with co-downregulated expression in three molting processes of different developmental stages (Figure 4B).

### 2.3. Different Expression Patterns of lncRNAs in Different Molting Processes from Larva to Adult

**Figure 5:**
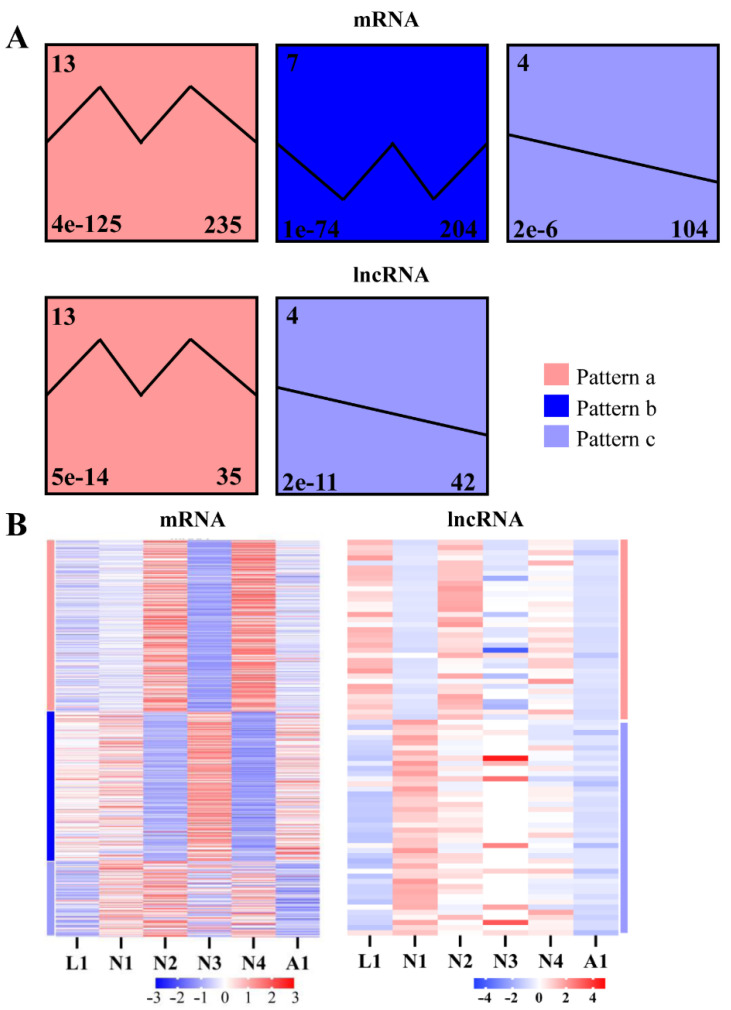
Different expression clusters of lncRNAs and mRNAs in the molting processes of different development stages. (**A**) Significant expression profiles (*p* < 0.05) of lncRNAs and mRNAs clustered via STEM software in the molting processes of different development stages. The numbers in the left upper part of the boxes are the profiles’ serial numbers, those in left lower part are *p*-values, and those in the right lower part are the number of transcripts contained in the profiles. Pattern a (positive zigzag pattern) is shown in red, Pattern b (negative zigzag pattern) is in blue, and Pattern c (sustained decreasing) is in violet. (**B**) Heat maps of lncRNAs and mRNAs in the expression pattern profiles. L1: late larva; N1: early protonymph; N2: late protonymph; N3: early deutonymph; N4: late deutonymph; A1: early adult.

To further investigate the expression dynamics of lncRNAs and mRNAs in three molting processes from larva to adult, the Short Time-series Expression Miner (STEM) was used to converge transcripts based on the changes in expression of lncRNAs and mRNAs. We established 20 models to analyze the expression dynamics of lncRNAs and mRNAs during the different molting processes from the larva to adult stages, but only 543 mRNAs and 77 lncRNAs converged into three and two expression clusters (*p* < 0.05), respectively (Figure 5B). In these clusters, expression Cluster 13 displayed a dynamic zigzag-like pattern (termed Pattern a), but expression Cluster 7 had the opposite expression pattern to expression Cluster 13 (termed Pattern b). Expression Cluster 4 had a sustained decline in all molting process from larva to adult (termed Pattern C). In total, 235 mRNAs and 35 lncRNAs were clustered in Pattern a, and 104 mRNAs and 42 lncRNAs were clustered in Pattern c. However, only 204 mRNAs and no lncRNAs were clustered in Pattern b (Figure 5A).

### 2.4. lncRNAs Involved in the Molting Processes of Different Developmental Stages

**Figure 6:**
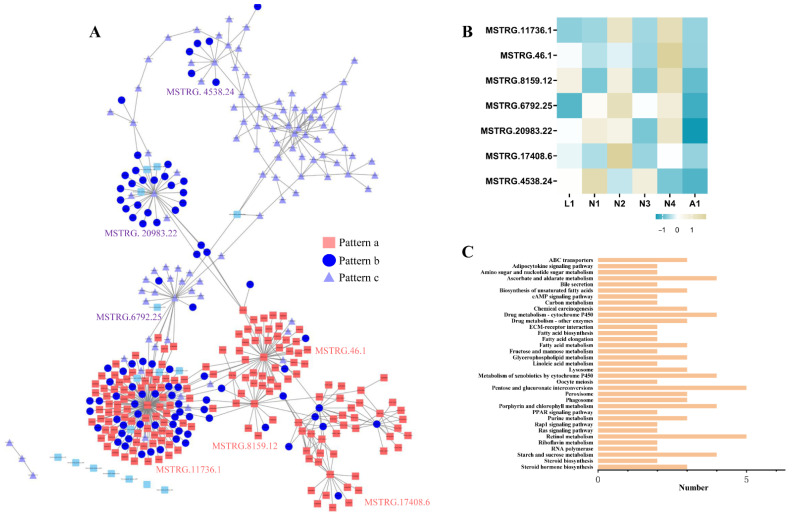
Functional prediction and identification of hub lncRNAs. (**A**) Co-expression networks of lncRNA and protein-coding genes constructed in the molting processes of different development stages by using Pearson’s correlation coefficient. (**B**) Heat map of seven hub lncRNAs in the molting process of different development stages. (**C**) KEGG pathways were enriched for each module in the co-expression network.

To identify the functions of lncRNAs in the molting processes of different development stages, the correlations of lncRNAs with mRNAs were analyzed by constructing co-expression networks. The correlation coefficients between lncRNAs and mRNAs in the expression clusters above were evaluated. In total, 69 lncRNAs were identified with a Pearson’s correlation coefficient of >|0.9| (Table S3). In the lncRNA–mRNA networks, an lncRNA with a higher number of connections with mRNAs was regarded as a hub lncRNA (Figure 6A). Seven hub lncRNAs were identified in the lncRNA–mRNA networks, and these were MSTRG.11736.1, MSTRG.46.1, MSTRG.8159.12, MSTRG.6792.25, MSTRG.20983.22, MSTRG.17408.6, and MSTRG.4538.24 (Figure 6B). The hub lncRNAs in the Pattern a module, including MSTRG.11736.1, MSTRG.8159.12, MSTRG.17408.6, and MSTRG.46.1, were the highest, implying that the lncRNAs in Pattern a had prominent function in the molting processes of different developmental stages. Among these hub lncRNAs, the degree values of MSTRG.11736.1 and MSTRG.46.1 were the highest, and these were considered as available candidates for regulating the molting processes of different developmental stages. 

The mRNAs with >0.9 correlation coefficients with the hub lncRNAs were analyzed by KEGG enrichment. The “retinol metabolism”, “Pentose and glucoronate interconversions”, “ascorbate and aldarate metabolism”, “steroid hormone biosynthesis”, “cytochrome P450”, “Porphyrin and chlorophyll II metabolism”, “starch and sucrose metabolism”, and “metabolism of xenobiotics by cytochrome P450” pathways were overrepresented in the top rank (Table S4). This indicated that hub lncRNAs play important roles in regulating steroid hormones, immunity, retinol, and sugar and xenobiotics metabolism. The fatty acid biosynthesis and metabolism pathways were enriched, implying that hub lncRNAs may be involved in fatty acid metabolism during the molting process. In addition, lncRNAs were related to molting-associated genes (Figure 6C). For example, lncRNA MSTRG.11736.1 was associated with UDP-glycosyltransferase genes (*UGT74*, *r* = −0.92; *UGT55*, *r* = −0.97; *UGT38*, *r* = −0.96) and cuticular protein-related genes (*CPAP*, r = 0.91; cuticular protein, *r* = 0.96; chitin-binding protein, *r* = 0.90). MSTRG.46.1was associated with ABC transporter genes (*ABCG-19*, *r* = 0.98; *ABCG-20*, *r* = 0.98) (Table 1 and Table S5).

## 3. Discussion

Molting is an important event in the development process of arthropods, which undergoes a series of physiological changes such as the forming and degradation of the cuticle, internal tissue apoptosis and remodeling, and other physiological processes [35]. lncRNA expression in the biological processes of development have been examined in insects [7,9]. In the mites, lncRNA has been identified in many physiological processes of mites such as *V. destructor* [33], *T. cinnabarinus* [34], and *Dermatophagoides farina* [36]. However, there is no previous report on the lncRNA involved in mite development and molting. To obtain an overview of lncRNAs in the molting processes from the larva to adult stages in the spider mite *P. citri*, we sequenced six development time points in the three molting processes from the larva to adult stages, including late larva (L1), early protonymph (N1), late protonymph (N2), early deutonymph (N3), late deutonymph (N4), and early adult (A1). The mRNAs had greater differential expression during the molting process than lncRNAs. The lncRNAs’ functions related to the molting processes in development were identified through co-expression networks between lncRNAs and mRNAs, and seven hub lncRNAs were strongly associated with molting-related genes. Based on the RNA-seq database, 9199 lncRNAs in total from all samples were identified, and a structural analysis of the lncRNAs showed that the lncRNAs tended to have shorter transcript lengths and have fewer exons compared with the protein-coding genes. These findings were similar to the lncRNA structure of insect species, such as *Zeugodacus cucurbitae* [37], *A. mellifera* [15], *T. castaneum* [9], and *S. furcifera* [8]. These results suggested that lncRNAs were less complex than mRNAs in *P. citri*. 

The differential expression analysis found that differentially expressed lncRNAs (386) were less than mRNAs (2944) during three molting processes. The temporal analysis uncovered fewer lncRNAs than mRNAs in the molting processes of different developmental stages. These results indicated that more mRNAs were involved in the molting process than lncRNAs in *P. citri*. According to the expression clusters of lncRNAs and mRNAs, lncRNAs followed concordant or opposite patterns to mRNAs during the molting processes of different developmental stages. These results suggested that lncRNAs could interact with mRNAs by affecting the expression of mRNAs to regulate molting-related signaling and proteins in the molting processes of *P. citri*. 

This molting process was regulated by multiple biological pathways such as: RNA transport, insect hormone biosynthesis, and other pathways [38]. In the molting processes of *P. citri*, lncRNAs could target multiple pathways, such as lysosome, RNA transport, and so on. To analyze the function of lncRNAs that could interact with mRNAs and nearby genes in the genome, the co-expression networks were constructed to identify the lncRNAs’ potential functions involved in the molting process. Co-expression analysis indicated that seven lncRNAs were regarded as hub lncRNAs. KEGG analysis of the target genes suggested that seven lncRNA targets were significantly enriched in steroid hormone biosynthesis, fatty acid metabolism, and ABC transporters. In steroid hormone biosynthesis of insects, UDP-glycosyltransferase as a horizontal transfer gene from a virus could inactivate ecdysone signaling, resulting in failure of the host larvae to molt in insects [39]. Nutrition has an important function in development and the molting process [40]. In the fatty acid metabolism pathway, the activity of fatty acid desaturases could assist the ecdysone signaling of development timing to regulate cuticle differentiation [41]. Fatty acid elongase has a mechanism that regulates cholesterol transportation and steroidogenesis through nutrition and development programs [42]. Other lncRNAs, MSTRG.11736.1 and MSTRG.46.1, were strongly associated with known genes, such as ABC transporter G member genes [43] and cuticular protein genes in the cuticular biosynthesis process [44], which are strongly involved in the molting process. Function annotation indicated that the lncRNA could target multiple pathways to regulate the molting process of *P. citri*. However, lncRNA not only targeted the mRNAs but also acted as miRNA precursors in current studies [45,46]. In insects, lncRNA could act as the precursors of miRNAs related to the ecdysone signaling pathway such as mir-8, mir-281, and mir-4 [9]. In our study, we only discussed the regulation relationship of lncRNA and mRNAs, and lacked information about the lncRNAs and miRNAs. Thus, we need to identify the lncRNAs related to miRNAs and verify the functions of these lncRNAs for mRNAs and miRNAs in the molting process in further studies.

## 4. Materials and Methods

### 4.1. Mites Culture 

*Panonychus citri* mites were obtained from the Banco orchard at the Citrus Research Institute, Southwest University (Chongqing, China). The strain was cultured on soybean leaves at a temperature of 27 ± 1 °C, a photoperiod of 14 h:10 h (L:D), and a relative humidity of 60 ± 10% in the laboratory.

### 4.2. RNA Extraction, cDNA Library Construction, and RNA Sequencing

Samples of mites in 8 h step-in and step-out molting processes from the larva to adult stage were collected following previously described methods [30], including the late larva (L1), early protonymph (N1), late protonymph (N2), early deutonymph (N3), late deutonymphs (N4) and early adult (A1) stages. Each development time point had 3 biological replicate samples. RNA was isolated by the TRIzol reagent (Invitrogen, Carlsbad, CA, USA) according to the protocol. The concentration and purity were determined using a Nanodrop 2000 spectrophotometer (Thermo Fisher Scientific, Wilmington, DE, USA), and the RNA integrity was assessed by the Agilent Bioanalyzer 2100 System (Agilent Technologies, CA, USA) with RIN > 8.0. Next, the rRNA from total RNA was removed using the Ribo-Zero rRNA Removal Kit (Epicentre, WI, USA). We used 1.5 μg RNA per sample to generate the sequencing libraries with the NEBNext Ultra Directional RNA Library Prep Kit for Illumina (NEB, Ipswich, MA, USA). Finally, the cDNA library for sequencing was constructed using the Illumina Hiseq platform.

### 4.3. lncRNA Target Prediction and Annotation

Prediction of the lncRNAs’ targets was based on *cis* function prediction. BEDTools v2.25.0 was used to screen the closest coding genes 10 kb upstream and downstream of the lncRNA. Next, the functional enrichment analysis of the target genes was performed by the DAVID (v6.7) database.

### 4.4. Transcriptome Assembly and Sequencing Data Analysis

The raw data quality was assessed using FastQC, and the raw data were cleaned to remove low-quality reads. The clean data were mapped to the spider mite *P. citri* reference genome (https://bioinformatics.psb.ugent.be/orcae/overview/Panci, , accessed on 22 June 2021) by the software tool HISAT 2.0. The transcriptome was assembled and annotated using the StringTie and gffcompare program based on the *P. citri* reference genome. The putative lncRNAs were screened from the unknown transcripts. The filter criteria and analysis pipeline of lncRNAs was as follows: (1) the known protein-coding transcripts were removed; (2) the transcripts longer than 200 bp in length with multiple exons and a FPKM more than 0.1 were included; (3) the transcripts containing class codes “i”, “e”, “x”, “u”, and “o” were included; (4) the transcripts with ORFs longer than 300 nt were filtered out; (5) the transcripts predicted to encode proteins were removed; and (6) the transcripts similar to protein-coding genes were removed. Finally, the residual transcripts were categorized as lncRNAs.

### 4.5. Differential Expression Analysis of lncRNAs and mRNAs

In order to identify the lncRNAs and mRNAs associated with the molting processes of different development stages in mites, RNA-sequencing (RNA-seq) libraries of transcriptome-wide gene expression profiles were constructed. The FPKMs of both lncRNAs and mRNAs in each sample were calculated by String Tie (v1.3.1). The differential expression analysis of all transcripts was performed by the DESeq R package (1.10.1). The transcripts with a *p*-value of <0.05 and a fold change of log_2_FC ≥ 1.0 were identified as differentially expressed between different molting processes.

### 4.6. Transcript Expression Dynamic Analysis of Different Molting Processes

The software Short Time-series Expression Miner (STEM, version 1.3.11) was applied to analyze the expression patterns of the differential expression transcripts in the molting processes from larva to adult. Twenty different expression clusters were set to generalize the expression changes of all transcripts in the molting processes of different development stages from larva to adult. The expressions of transcripts were normalized in every molting time point before being clustered by log_2_(FPKM). According to a correlation coefficient of *r* > 0.7, the mRNAs or lncRNAs were divided into the model clusters that conformed to their expression patterns. The clusters were considered as significant expression clusters with a *p*-value of <0.05.

### 4.7. Co-Expression Analysis between lncRNAs and mRNAs in Molting Processes

The correlation coefficients of mRNAs and lncRNAs in the time series expression patterns were assessed. Pearson’s correlations of the lncRNAs and mRNAs were determined using R software. The lncRNAs and mRNAs with correlation coefficients of |*r*| > 0.9 were considered as co-expression genes. The correlation coefficients between mRNAs and lncRNAs were used as input in the software Cytoscape (v3.6.1) to construct a co-expression network.

### 4.8. KEGG Pathway Analysis of Co-Expressed mRNAs

We used the DAVID database to determine the functional annotation enrichment analysis of co-expressed mRNAs in KEGG pathways. The enrichments with *p*-values < 0.05 were considered as statistically significant.

## 5. Conclusions

This study demonstrated the potential relationships between lncRNAs and mRNAs during the molting process of different developmental stages in mites, and the potential functions of lncRNAs in regulating the molting processes of mite development. 

## Figures and Tables

**Table 1:** Correlations between known molting-related genes and lncRNAs.

lncRNA	Transcript	*r*	Annotation
MSTRG.11736.1	EVM0000696	−0.92326	UDP-glycosyltransferase 74
MSTRG.11736.1	EVM0001593	−0.96779	UDP-glycosyltransferase 55
MSTRG.11736.1	EVM0006429	−0.95658	UDP-glycosyltransferase 38
MSTRG.11736.1	EVM0004344	−0.94306	UDP-glycosyltransferase
MSTRG.11736.1	EVM0006915	0.905254	cuticular protein
MSTRG.11736.1	EVM0006682	0.956653	cuticular protein
MSTRG.11736.1	EVM0005426	0.900242	Chitin binding protein peritrophin-A
MSTRG.46.1	EVM0001502	0.980124	ABCG-20
MSTRG.46.1	EVM0002824	0.979736	TuABCG-19

## Data Availability

Data is contained within the article or supplementary material.

## Supplementary Materials

The following are available online at https://www.mdpi.com/article/10.3390/ijms22136909/s1.
