# Supplementary material for: Genome-Wide Characterization and Identification of Long Non-Coding RNAs during the Molting Process of a Spider Mite, Panonychus citri
Source: Int J Mol Sci. 2021 Jun 27;22(13):6909. doi: 10.3390/ijms22136909 (PMC8269015; doi:10.3390/ijms22136909)
Supplement: Supplementary file 1 [file ijms-22-06909-s001.zip › Table S1.pdf]

Table S1 Summary statistics of the RNA-seq data

Sample ID	Read Sum	Base Sum	GC (%)	N (%)	Q30 (%)	Genome mapping rate (%)
L1-1	62,273,457	18,627,717,550	37.91	0	95.26	92.79
L1-2	62,080,354	18,548,483,096	38.08	0	95.34	92.62
L1-3	62,672,582	18,631,196,222	37.99	0	95.47	92.22
N1-1	60,736,392	17,967,258,808	38.57	0	95.42	88.63
N1-2	59,094,611	17,647,165,578	38.95	0	95.18	84.85
N1-3	65,601,551	19,457,062,924	38.41	0	95.62	88.41
N2-1	55,548,267	16,576,659,110	39.72	0	95.87	84.75
N2-2	57,745,049	17,076,506,940	39.68	0	95.97	89.92
N2-3	62,422,669	18,607,072,334	38.83	0	95.71	83.47
N3-1	77,506,059	23,004,868,274	39.47	0	95.8	82.70
N3-2	86,209,009	25,683,992,226	39.16	0	95.63	84.66
N3-3	85,202,276	25,450,234,840	38.83	0	95.62	74.61
N4-1	84,374,619	25,176,637,398	38.24	0	95.58	88.08
N4-2	84,592,547	25,194,442,380	38.67	0	95.65	87.65
N4-3	89,806,072	26,835,940,438	38.61	0	95.54	77.22
A1-1	60,784,438	18,107,891,686	39.04	0	95.67	84.66
A1-2	63,794,080	19,029,795,574	39.28	0	95.69	80.12
A1-3	59,414,638	17,730,275,700	42.47	0	95.59	81.75

Read Sum: pair-end reads in clean data. Base Sum: the total base numbers in clean data. GC: the percentage of G and C in clean data. N: percentage of unidentified bases in clean data. Q30: nucleotides with a quality value above 30 in reads. L1: late larva; N1: early protonymph; N2: late protonymph; N3: early deutonymph; N4: late deutonymph; A1: early adult.
